# Hospital Reports—New York Woman’s Hospital

**Published:** 1876-05

**Authors:** 


Hospital Reports—New York Woman's Hospital.—*Uterine Polypus.*—A patient, aged forty-nine, entered Dr. Emmet's service, saying that she had been under treatment for falling of the womb. There had been no bloody discharge from the vagina for eighteen months, and when she was placed on the examining-table a mass of the size of an almond was found to be attached to the posterior lip of the cervix. There was no distress occasioned by the growth. Treatment—The patient was anæsthetized, and then the uterus drawn down by means of a tenaculum and a slip-noose placed around the pedicle, and the growth cut off with the scissors. The after-treatment consisted in making an application of Churchill's tincture of iodine every few days. After ten days the patient was completely recovered.

*Fungoid Granulations of the Mucous Membrane of the Uterus.*—A patient, aged forty-six, single, entered the hospital with the following history: Began menstruating at fifteen, and had been regular till three months before admission. At that time failed to notice any vaginal flow for six weeks, but when menstruation began it continued, without ceasing, for three weeks.

After admission to hospital the cervix uteri was dilated by means of sponge tents sufficiently large to introduce the index-finger. It was then found that several mucous polypi could be discovered. They were removed by Dr. Emmet, and Churchill's tincture of iodine applied to the cavity of the uterus.

For six months after this treatment the patient was regular, but at the end of that time menorrhagia returned. The cervix uteri was again dilated, and one drachm of granulations removed. Shortly after this operation the patient, contrary to orders, got out of bed. Symptoms of pelvic cellulitis soon after developed, but were readily subdued by vaginal injections of hot water. Convalescence was soon established, and the patient discharged from the hospital.

*Intra-Uterine Fibroid.*—A patient, aged thirty-four, entered hospital with the following history: She was married when twenty-five years of age, and since that time has had four children and three abortions. Four years ago had a polypus expelled by the contractile efforts of the uterus. For the past three years has had dysmenorrhœa throughout the flow. Latterly this has increased, and for the past four months menstruation has continued sixteen days. Physical Examination—When the finger was introduced a tumor was detected, filling the cavity of the cervix and protruding into the vagina. On more thorough examination, the pedicle was found attached to the posterior surface of the uterus. On introduction of Sims' speculum, the surface of the mass was found to be irregular and shining, showing it to be a fibrous tumor. Operation—The patient was anæsthetized and placed on her back. A noose with slip-knot was then placed around the pedicle by Dr. Emmet, and traction made. The pedicle was found to be attached to the fundus posteriorly. After traction was made for a few moments, the pedicle was cut and the growth removed. The after-treatment consisted in uterine injections of hot water, followed by the injection of two drachms of Churchill's tincture of iodine. After this application all hemorrhage ceased. A pledget of cotton soaked in glycerine was placed against the cervix, and the vagina slightly tamponed. Two days after, the dressing was removed and the uterus syringed out with hot water. No fever followed the operation, and in two weeks afterward the patient was discharged cured. *Medical and Surgical Reporter.*

